# Supplementary material for: Novel Nile Blue Analogue Stains Yeast Vacuolar Membrane, Endoplasmic Reticulum, and Lipid Droplets, Inducing Cell Death through Vacuole Membrane Permeabilization
Source: J Fungi (Basel). 2021 Nov 16;7(11):971. doi: 10.3390/jof7110971 (PMC8623074; doi:10.3390/jof7110971)
Supplement: Supplementary file 1 [file jof-07-00971-s001.zip › jof-1469516-supplementary.pdf]

Journal of *Fungi*

MDPI

Supplementary Materials

Novel Nile Blue Analogue Stains Yeast Vacuolar Membrane, Endoplasmic Reticulum and Lipid Droplets, Inducing Cell Death through Vacuole Membrane Permeabilization

João Carlos Canossa Ferreira [1,2,3,*], Carla Lopes [1], Ana Preto [1,2], Maria Sameiro Torres Gonçalves [3] and Maria João Sousa [1,2,*]

[1] Centre of Molecular and Environmental Biology (CBMA), Department of Biology, Campus of Gualtar, University of Minho, 4710-057 Braga, Portugal; carla.lopes89@hotmail.com (C.L.); apreto@bio.uminho.pt (A.P.)
[2] Campus of Gualtar, IBS-Institute of Science and Innovation for Bio-Sustainability, University of Minho, 4710-057 Braga, Portugal
[3] Centre of Chemistry, Department of Chemistry, Campus of Gualtar, University of Minho, 4710-057 Braga, Portugal; msameiro@quimica.uminho.pt
* Correspondence: id7351@uminho.pt (J.C.C.F.); mjsousa@bio.uminho.pt (M.J.S.)

Captions:

Figure S1 Effect of BaP1 on mitochondrial membrane potential.

Figure S2 Effect of BaP1 on ROS production.

Figure S3 Effect of BaP1 on intracellular calcium accumulation.

Figure S4 A and B Evaluation of DNA fragmentation and DNA cell content.

Figure S5 Effect of BaP1 on plasma membrane integrity.

Figure S6 BaP1 intracellular distribution in lipid droplets mutants.

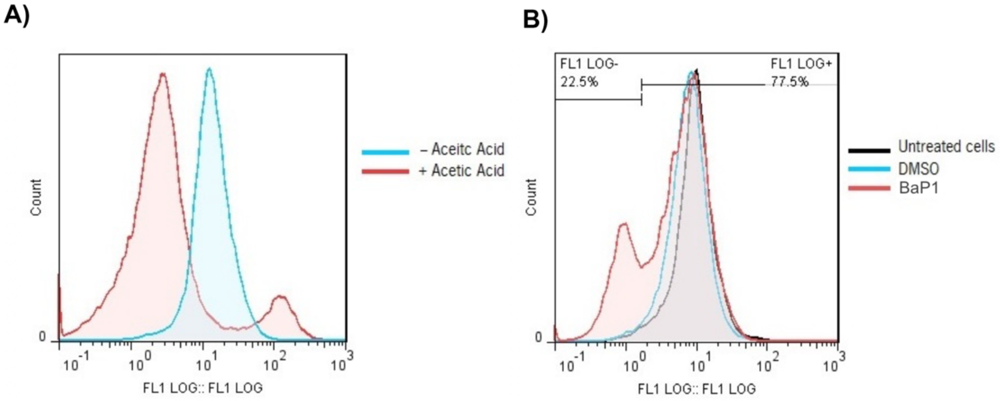

Figure S1. Effect of BaP1 on mitochondrial membrane potential. A) Effect of acetic acid (150 mM at pH 3.0 (positive control)) on *S. cerevisiae* BY4741 mitochondrial membrane potential. For flow cytometry analysis, the cells were collected before (- acetic acid) and after 60 minutes of acetic acid treatment (+ acetic acid) and stained with 0.24 μM of $Dioc_6(3)$. B) Effect of BaP1(300 μM) and DMSO (0.35%) on BY4741 mitochondrial membrane potential. The represented results correspond to the 60 minutes time point. Results are expressed as a mono-parametric histogram of $Dioc_6(3)$ fluorescence representing one of three independent experiments. Gate defined by auto-fluorescence sample.

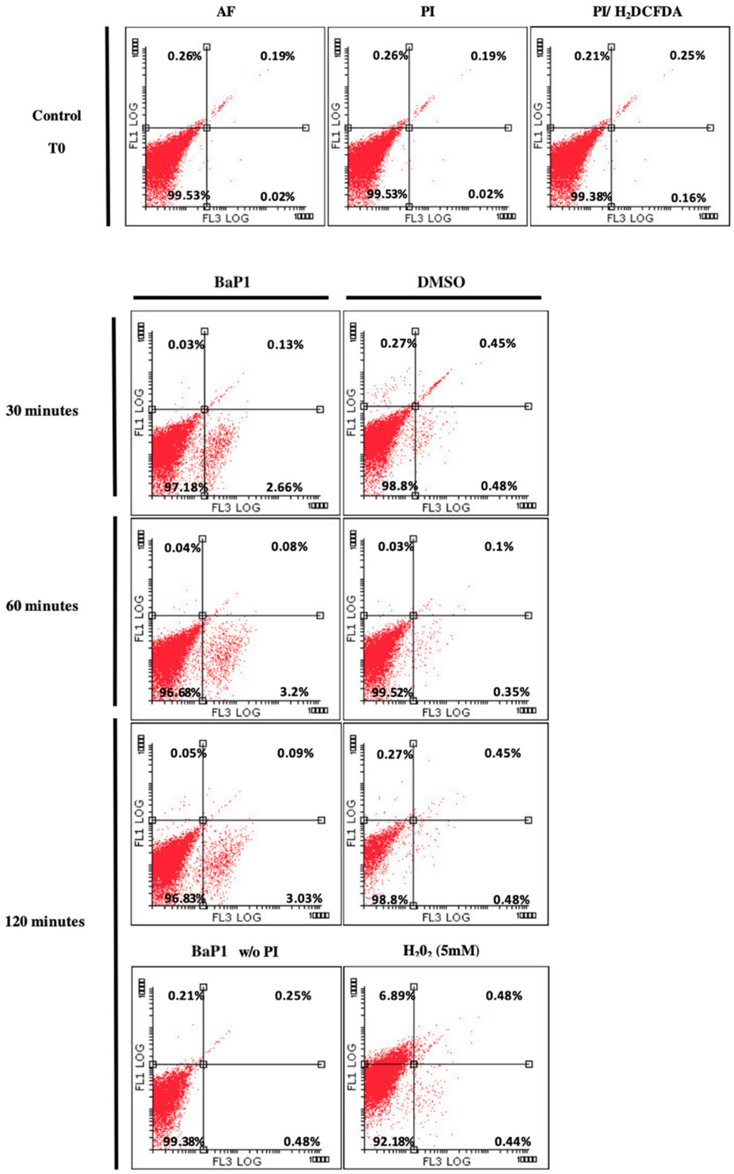

Figure S2. Effect of BaP1 on ROS production. Representative experiment of the effect of BaP1(300 μM) on *S. cerevisiae* W303-1A ROS production. For flow cytometry analysis the cells were collected at different time points and stained with 40μg/mL H_2DCFDA and 2 μg/mL of PI. 5mM Hydrogen peroxide (H_2O_2) was used as positive control.

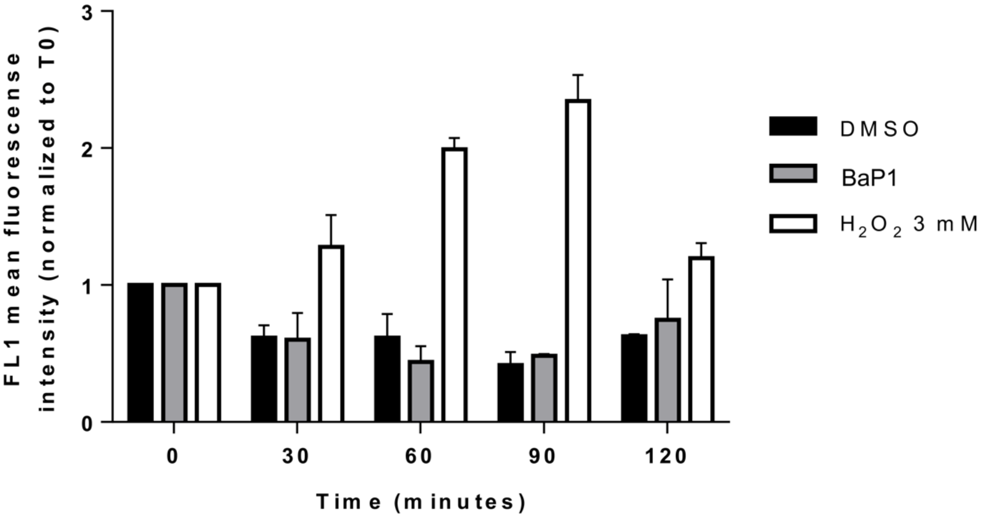

Figure S3. Effect of BaP1 on intracellular calcium accumulation. Effect of BaP1 (300 μM), DMSO (0.35%) and H2O2 (3 mM at pH 3.0 (positive control)) on *S. cerevisiae* BY4741 intracellular calcium accumulation. For flow cytometry analysis the cells were collected at different time points and stained with 5 μM FLuo4-AM. Results are expressed as ratio values estimated by dividing the FL1 mean fluorescence intensity of each sample by the FL1 mean of the time 0 sample. Statistical analysis: BaP1:(T0 vs T30) ns, (T0 vs T60) ns, (T0 vs T90) ns and (T0 vs T120) ns; DMSO: (T0 vs T30) ns, (T0 vs T60) ns, (T0 vs T90) ns and (T0 vs T120) ns; H2O2: (T0 vs T30) ns, (T0 vs T60)**, (T0 vs T90)*** and (T0 vs T120) ns. Values are means with SD (n≥2). Statistical analysis was performed by two-way ANOVA. ns non-significant, *P<0.05, **P<0.01, ***P<0.001, ****P<0.0001.

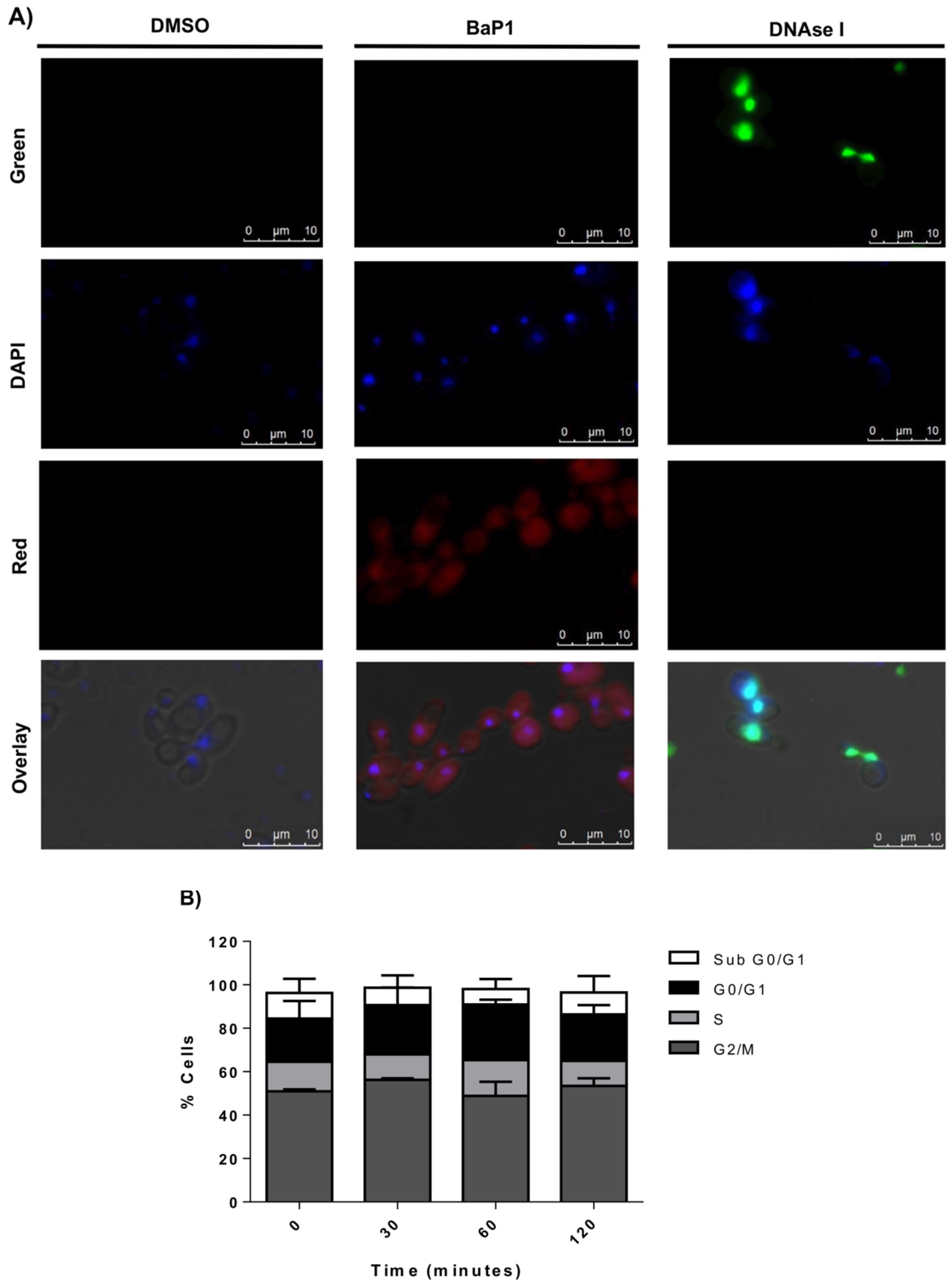

Figure S4. Evaluation of DNA fragmentation and DNA cell content A) Florescence microscopy images of TUNEL assay of *S. cerevisiae* BY4741 after 30 minutes treatment with BaP1 (300 μM) and DMSO(0.35%). Samples were collected at different time points and subjected to TUNEL protocol. B) Effect of BaP1 (300 μM) and DMSO (0.35%) in cell cycle of *S. cerevisiae* W303-1A. Cells were stained with SYTOX Green and analyzed by flow cytometry. Values are means with SD (n≥2). Scale bar = 10 μm.

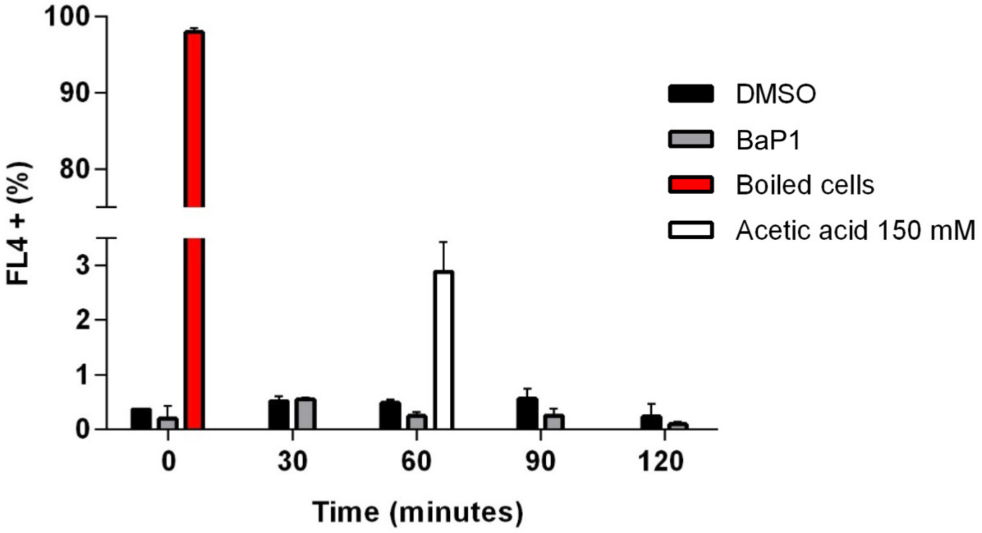

Figure S5. Effect of BaP1 on plasma membrane integrity. Effect of BaP1 (300 μM), DMSO (0.35%) and acetic acid (150mM at pH 3.0) on propidium iodide (PI) staining of *S. cerevisiae* BY474. Boiled cells were used as positive control for PI staining. For flow cytometry analysis the cells were collected at different time points and stained with 2μg/mL of PI. Results are expressed as percentage of FL4 positive population (FL4 LOG +) Statistical analysis: BaP1: (t0 vs t30)ns, (t0 vs t60)ns, (t0 vs t90) ns, (t0 vs t120) ns; DMSO: (t0 vs t30) ns, (t0 vs t60) ns, (t0 vs t90) ns, (t0 vs t120) ns; Acetic acid: (t0 vs t60)****. Values are means with SD (n≥2). Statistical analysis was performed by two-way ANOVA. ns non-significant, *P<0.05, **P<0.01, ***P<0.001, ****P<0.0001.

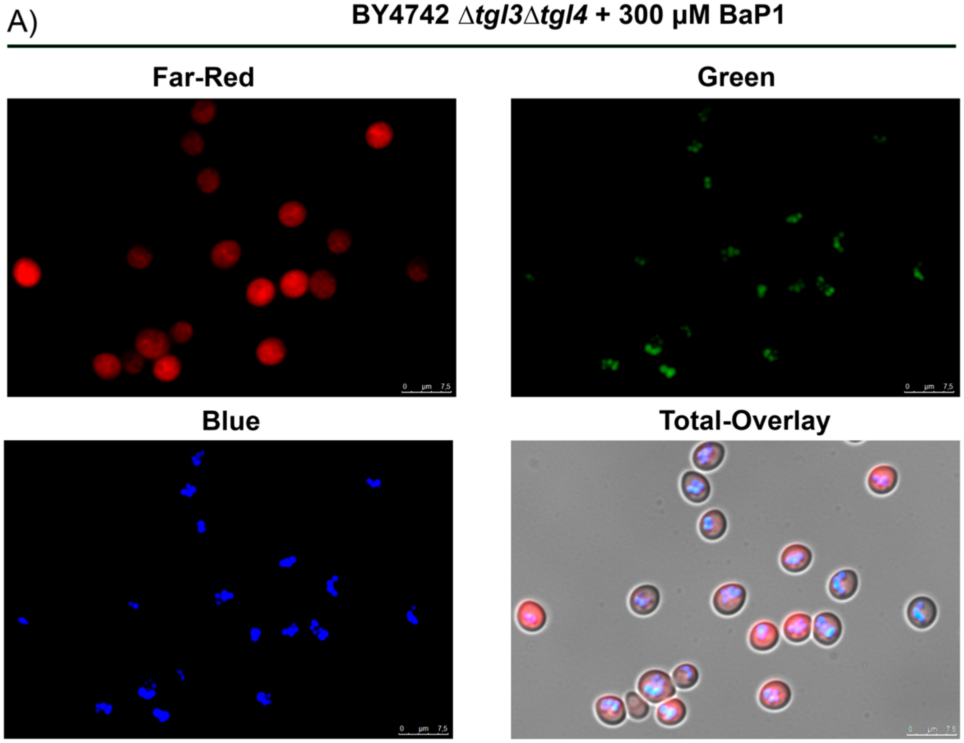

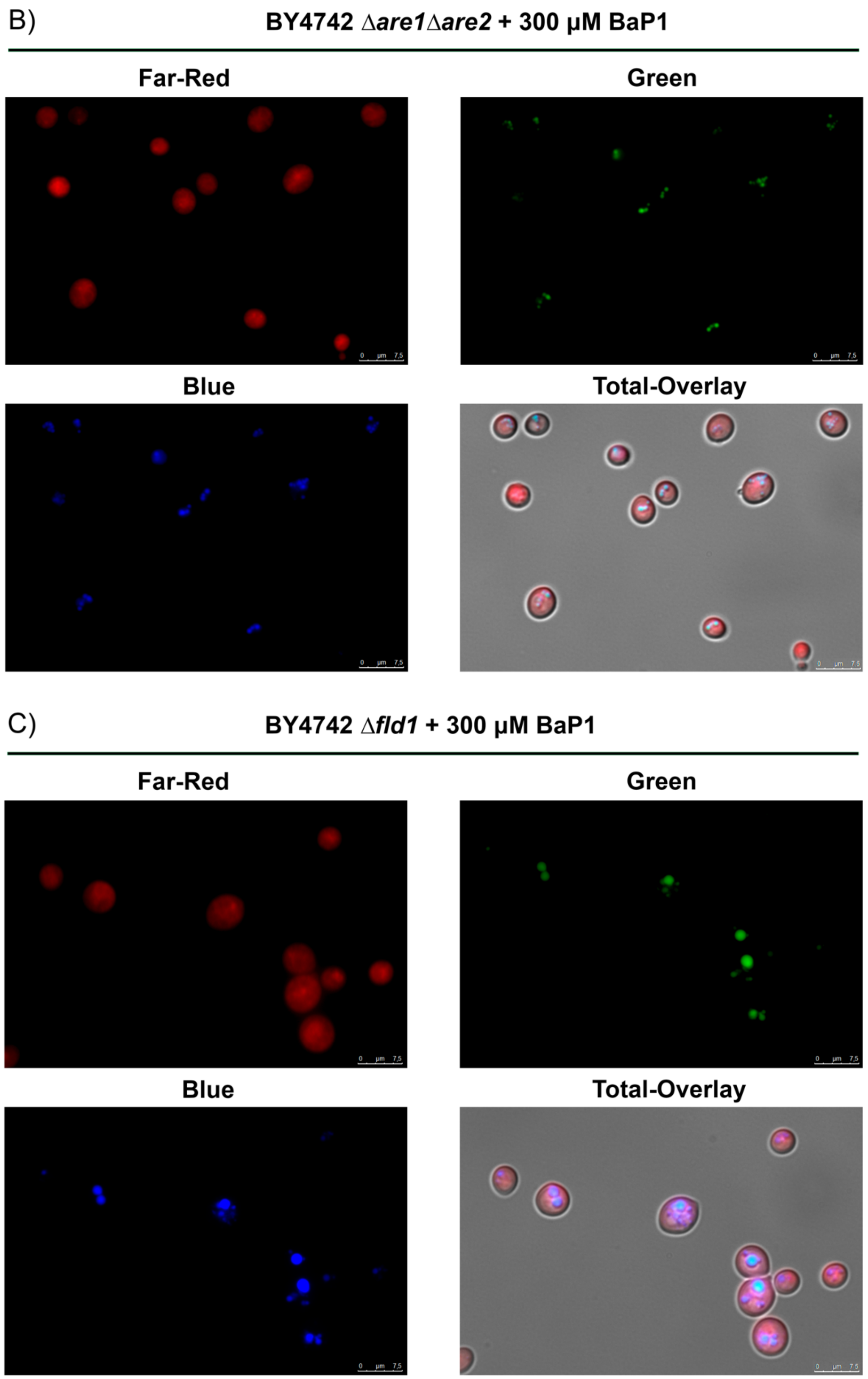
B)
BY4742 ∆are1∆are2 + 300 µM BaP1
Far-Red
Green
Blue
Total-Overlay
C)
BY4742 ∆fld1 + 300 µM BaP1
Far-Red
Green
Blue
Total-Overlay

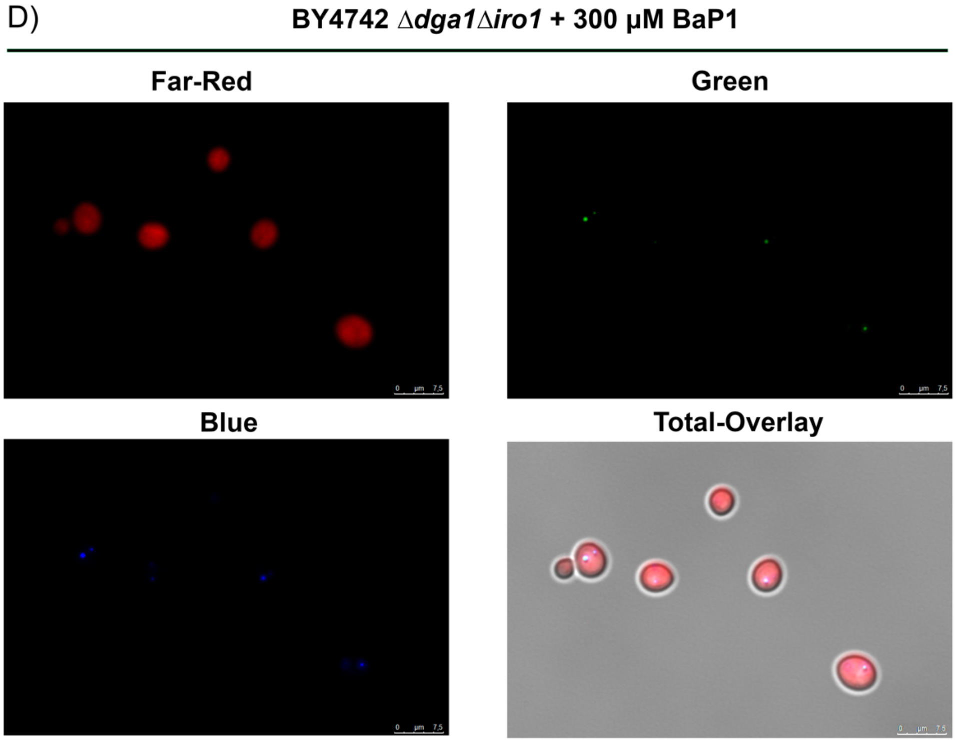

Figure S6. BaP1 intracellular distribution in lipid droplets mutants. A) Florescence microscopy images of BY4742 *Δtgl3Δtgl4* cells after incubation with BaP1 (300 µM). B) Florescence microscopy images of BY4742 *Δare1Δare2* cells after incubation with BaP1 (300 µM). C) Florescence microscopy images of BY4742 *Δfld1* cells after incubation with BaP1 (300 µM). D) Florescence microscopy images of BY4742 *Δdga1Δiro1* cells after incubation with BaP1 (300 µM). BY4742 *Δtgl3Δtgl4* (lacking the major TAG lipases, which exhibit higher LD accumulation), BY4742 *Δare1Δare2* (lacking the steryl ester synthases and thus harbors LDs that contain triacylglycerols only), BY4742 *Δfld1* (lacking the yeast ortholog of seipin, resulting in a fewer and larger LDs) and BY4742 *Δdga1Δiro1* (mutant lacking acyl-CoA and phospholipid dependent diacylglycerol (DAG) acyltransferases and thus harbors LDs that contain steryl esters only). Samples were stained in YPD at room temperature and visualized by epifluorescence microscopy after 5 minutes with a 100x oil immersion objective.
